# Clinical and functional characterization of p.Lys322stop variant in the *SERPINC1* gene causing severe thrombophilia

**DOI:** 10.1186/s13023-024-03498-y

**Published:** 2024-12-20

**Authors:** Haiyue Zhang, Xinyang Yue, Tenglong Dai, Jun Wu

**Affiliations:** 1 Thrombosis Research Center, Beijing Jishuitan Hospital, Capital Medical University, Xicheng District, Beijing, 100035 China; 2 Department of Clinical Laboratory, Peking University Fourth School of Clinical Medicine, Xicheng District, Beijing, 100035 China

**Keywords:** *SERPINC1*, VTE, Antithrombin deficiency, Recombinant AT protein

## Abstract

**Background:**

Identification of mutations in the *SERPINC1* has illuminated the intricate pathways underlying antithrombin (AT) deficiency. Our group identified a variation in the *SERPINC1* gene (c.964 A > T, p.Lys322stop) and further investigated the mechanism of this variant causing AT deficiency.

**Methods:**

Multiple in silico tools were utilized to predict the conservation of mutations and their impact on the AT structure. The coagulation state was evaluated using the thrombin generation assay. Recombinant AT was overexpressed in HEK293T cells. Intracellular kinetics and extracellular secretion of recombinant AT-K322* were scrutinized by RT-qPCR, Western blotting, ELISA, and immunocytofluorescence.

**Results:**

Analysis of conservation in silico indicated 43 out of the 143 amino acids deleted byAT-K322* in AT were highly conserved across homologous species. In vitro expression experiments showed that there was no significant difference in mRNA levels between the mutant (AT-K322*) and wild-type (AT-WT) forms of the protein. The truncated AT-K322* protein was clearly detected in cell lysates, but not in the culture medium.

**Conclusion:**

AT-K322* resulted in the generation of a truncated protein, which in turn affected the secretion of AT, ultimately leading to AT deficiency.

**Supplementary Information:**

The online version contains supplementary material available at 10.1186/s13023-024-03498-y.

## Introduction

Antithrombin (AT) is the main component of physiological anticoagulant substances in the body [1, 2], accounting for approximately 70% of the total activity of the anticoagulant system. It consists of three β-sheets (A to C), nine α-helices (A to I), and a reactive center loop (RCL). The gene encoding AT protein is located on human chromosome 1q23−25, with a total length of 13.4kb [3], consisting of 7 exons and 6 introns. Among the structural features crucial for the inhibition of coagulation proteases, the RCL and the D-helix are the most extensively studied and play pivotal roles. The RCL is a segment of an exposed stem-loop structure that comprises twenty residues, spanning from P15 to P5′. When the RCL binds to the active site of a serine protease, it triggers the cleavage of the peptide bond between the P1 arginine and P1′ serine residues [4]. This interaction leads to a significant conformational change in the RCL, effectively trapping the protease in an inactive, acylated complex [5]. This covalent inactivation neutralizes the targeted protease. By contrast, the D-helix lacks a direct interactive site, but it contains several basic residues that bind to heparin, thereby accelerating the reactivity of the serpin with coagulation proteases by a factor of 1000-fold [4].

Hereditary AT deficiency, a common autosomal dominant genetic disorder caused by mutations in the *SERPINC1* gene, is recognized as the most severe form of inherited thrombophilia [6]. Compared to the general population, individuals with AT deficiency face a heightened risk of VTE, with the peril escalating up to 14 times [7]. The prevalence of AT deficiency within the population varies between 1:500 to 1:3000, yet amongst those suffering from VTE, the prevalence may soar to as high as 2–5% [8]. It is classified as type I (low activity and low antigen) and type II (low activity and normal antigen) deficiency [9]. The latter can be further sub-classified based on the location and functional consequences of the defect (heparin binding site, reactive site and pleiotropic effect) [10].

To date, more than 500 mutations causing hereditary AT deficiency have been documented. It is worth noting that there are few polymorphisms found in the *SERPINC1* gene, mainly located in introns, which reflects the molecule’s susceptibility to subtle alterations in its function or structure [11]. We present a rare AT-deficient case carrying a natural mutant (c.964 A > T), which was initially reported by our laboratory [12]. The impairment of AT is confirmed through the recombinant expression of the mutant.

## Materials and methods

### Patient

A 24-year-old young male was diagnosed with type I AT deficiency (AT antigen, 49 mg/L; AT activity, 50%). The proband presented deep venous thrombosis of the right leg with pulmonary embolism without any identifiable inducement. Despite the poor response to heparin-calcium injection therapy, his symptoms treated with rivaroxaban. None of the relatives had been exposed to the risk factors associated with thrombosis, nor did any of them have a previous history of thrombosis.

### Coagulation screening

Peripheral venous blood samples were then collected from the proband and his family members using anticoagulation tubes with 109 mmol/L trisodium citrate. Following centrifugation, the upper layer of plasma was used for routine coagulation assays and other research. Protein C activity (PC: A) and AT: A were assessed utilizing a commercial kit using the chromogenic substrate method on the Stago STA-Max automatic blood analyzer (Diagnostica Stago, Asnieres sur Seine, France). Protein S activity (PS: A) was determined using the clotting method. The AT: Ag was measured by ELISA assay (Jianglai, China). The study was approved by the Ethics Committee of the Beijing Jishuitan Hospital, with all participants providing informed consent.

### Screening for other risk factors

A comprehensive assessment of additional thrombosis risk factors was conducted for the proband and other family members, including analysis of factor V Leiden mutation (G1691A), prothrombin G20210A mutation, and antiphospholipid antibodies.

### Mutation analysis of the AT-deficient family

The DNA extraction, PCR, and direct sequencing were conducted following established protocols [13]. According to the original *SERPINC1* sequence documented in the NCBI gene bank (NC_000001.11), potential genetic variations were identified using the Chromas software. The position of amino acids was shown after signal peptide cleavage.

### Thrombin generation assay (TGA)

The calibrated automated thrombogram technique was utilized to perform the TGA in platelet-poor plasma (PPP), utilizing a Fluoroskan Ascent FL reading meter (Thermo Fisher Scientific, Massachusetts, USA) and the commercial kits (Thrombinoscope BV, Rijswijk, Netherlands). PPP was used to assess lag time, endogenous thrombin potential (ETP), peak, and time to peak (ttPeak) to quantify thrombin generation among study participants. The control group consisted of PPP from 10 healthy individuals.

### In silico analysis

The ClustalX−2.1-win software was utilized to evaluate the degree of conservation in Homo sapiens and 9 other homologous species: Mus musculus, Rattus norvegicus, Pan troglodytes, Macaca mulatta, Canis lupus familiaris, Bos taurus, Xenopus tropicalis, Gallus gallus, and Danio rerio. A molecular model of AT was constructed based on the intricate three-dimensional structure of AT (PDB: 1azx). The mutation was modeled using PyMol software and Swiss-Pdb Viewer software.

### Construction of recombinant AT expression vector

The pCDH-copGFP-T2A-Puro plasmid vector was utilized for in vitro studies on gene over-expression. Primers were designed based on the coding region of the target sequence *SERPINC1*. The purified PCR products were inserted into the plasmid vector (pCDH-copGFP-T2A-Puro; SBI, California, USA). Recombinant expression vectors of the variant were formed using the Mut Express II Fast Mutagenesis Kit (Vazyme, Nanjing, China) and mutation-inducing primers. The constructed wild-type and mutant plasmid vectors sequences were confirmed.

### Recombinant AT expression

Under 5% CO_2_ at 37℃, HEK293T cells were incubated in Dulbecco’s Modified Eagle Medium (DMEM, Gibco, USA) supplemented with 10% fetal bovine serum (FBS, Gibco, USA), 100 U/ml penicillin, and 0.1 mg/ml streptomycin (Beyotime, Shanghai, China) for cell growth. Expression vectors were introduced into HEK293T cells using Lipofectamine^®^ 3000 Reagent (Invitrogen, Carlsbad, CA, USA) following the manufacturer’s instructions.

### Total RNA extraction and quantitative real-time PCR (RT-qPCR)

After 48 h of recombinant AT expression vector transfection into HEK293T cells, the transfected cells were harvested. Total RNA was extracted utilizing RNAiso Plus reagent (Takara Bio, Kyoto, Japan) and RNA was reverse transcribed to cDNA with the Thermo Scientific RevertAid RT SuperMix for qPCR kit (Thermo Scientific, California, USA), in accordance with the guidelines provided by the manufacturer. RT-qPCR was performed using the Applied Biosystems SYBR qPCR Master Mix kit (Applied Biosystems, California, USA) on a PCR thermocycler (ThermoFisher Scientific, MA, USA). The primers were designed by Primer-BLAST (5′-GGAAGGAACTGTTCTACAAGGC−3′) and glyceraldehyde−3-phosphate dehydrogenase (GADPH) gene was chosen as the reference gene. Data analysis was conducted utilizing the comparative ΔΔCt method.

### Western blotting (WB) and ELISA

After 6 ~ 8 h of transfection, the cell culture medium was replaced with FBS-free DMEM. After 48 h, a part of the culture medium and the adherent cells were collected. The culture medium underwent centrifugation at 4,000 g for 30 min in an Amicon Ultra 30 k ultrafiltration centrifuge tube (Merck Millipore, Darmstadt, Germany). Then, the cell supernatant concentrate was collected. The cell samples were lysed with Radio Immunoprecipitation Assay (RIPA, NCM, Suzhou, China) and AT: A of concentrated cell supernatant was detected on the Stago STA-Max automatic blood analyzer. The quantified culture media and cell lysates were analyzed by 10% SDS-PAGE. After protein transfer, blocking, and antibody incubation, protein blotting was performed using the ECL ultra-sensitive chemiluminescent detection kit (Millipore, Burlington, USA). AT and GADPH mouse anti-human IgG monoclonal antibody (Proteintech, Chicago, USA) were used as the primary antibody, while the secondary antibody chosen was a goat anti-mouse IgG conjugated with horseradish peroxidase (HRP) (Beyotime, Shanghai, China). AT proteins in culture supernatants and cell lysates were quantified through the use of the Human Antithrombin-III ELISA kit (Jianglai, China), and the relative AT: Ag was determined by setting wildtype AT at 100%.

### Immunofluorescence (IF)

After a 48 h transfection of plasmids, cells were fixed with 4% paraformaldehyde for 20 min. Permeabilization was then carried out using Triton X−100 (Beyotime, Shanghai, China) and incubated at room temperature for 20 min. Subsequently, the cells were blocked with PBS containing 5% bovine serum albumin for 30 min at room temperature. Following this, mouse anti-human AT IgG monoclonal antibody (Proteintech, 1:500 dilution, 4℃ for 12 h) and Cy3-labeled goat antimouse antibody (Proteintech, 1:100 dilution, 37℃ for 2 h) were successively added. Finally, an amount of antifluorescence quenching reagent containing DAPI (ThermoFisher Scientific, MA, USA) was added. IF imaging was promptly captured using Olympus Imaging Systems (Olympus, Tokyo, Japan).

## Results

### The laboratory tests and mutations in *SERPINC1*

**Fig. 1:**
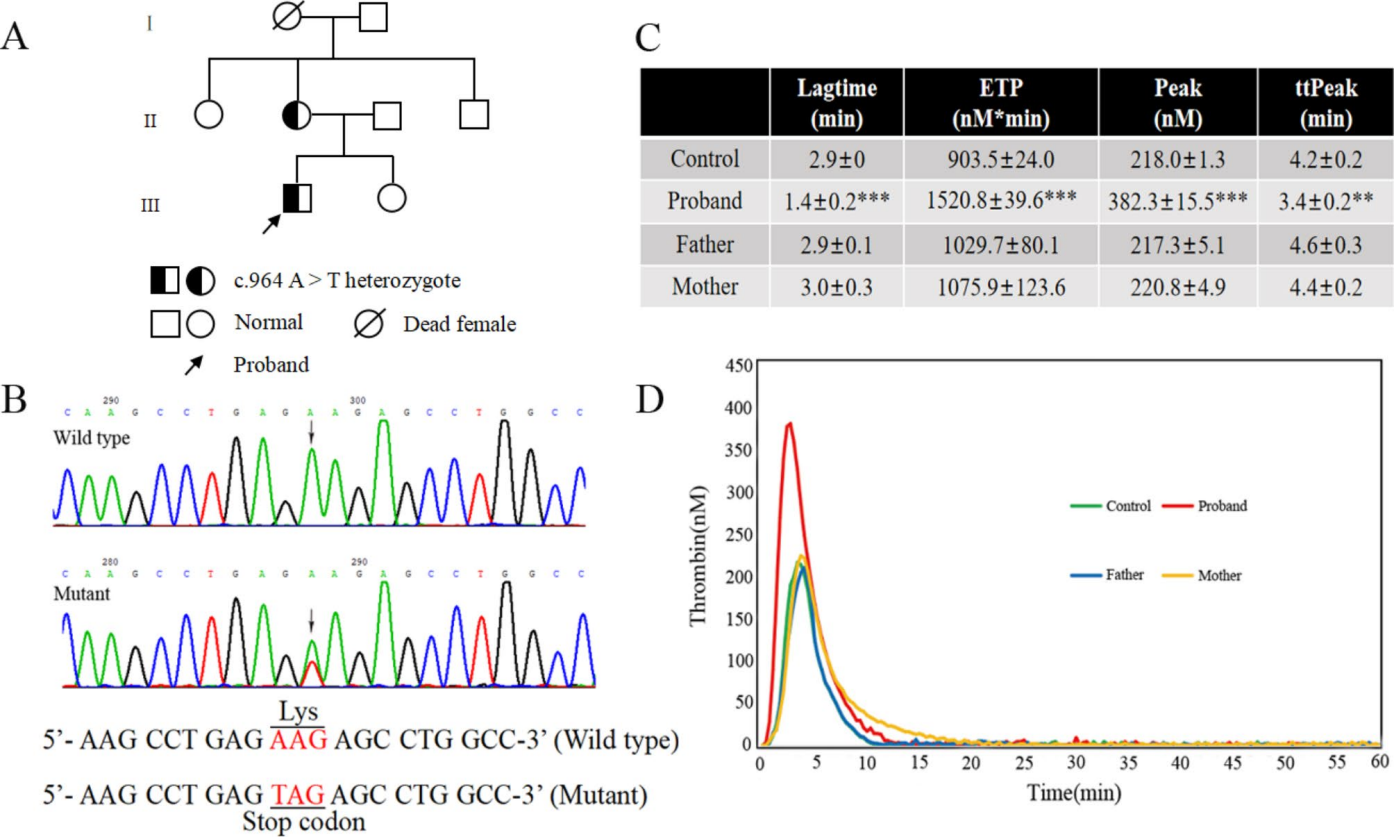
Pedigree analysis and genetic testing in a family with AT deficiency. (**A**) Pedigree of the AT-deficient family. (**B**) Sequencing diagram of c.964 A > T. From top to bottom: direct sequencing of wild type and direct sequencing of mutant. (**C**) Specific values of four parameters of the TGA in four subjects. (**D**) Curves of TGA. Four colors represent the four subjects

A total of 7 members from three generations were recruited for this investigation (Fig. 1A). The basic laboratory tests of the family are presented in Table 1. Laboratory investigations into thrombophilia in the proband unveiled a significant reduction in AT: A, with levels at 50%. The proband denied a history of smoking, surgery, and liver or kidney disease. Upon genetic scrutiny of the *SERPINC1* gene, it was discovered that the proband carried a mutation in exon 5, specifically the c.964 A > T (Fig. 1B), leading to the premature termination of the AT protein (p.Lys322stop). Further examination of the family lineage revealed that the proband’s mother exhibited roughly half the normal AT: A level and molecular genetic testing confirmed she carried the c.964 A > T heterozygous mutation. Individuals within the family possessing this mutation all showed a similar decrease in AT: A, indicating a diagnosis of hereditary type I AT deficiency.

**Table 1:** Clinical manifestations and coagulation test results of the AT-deficient family

Family mumber	Age	PC: A (%)	PS: A (%)	AT: A (%)	AT: Ag (mg/L)
Proband	24	116	88.5	50	49
Grandfather	76	105	97.1	102	100
Aunt	48	95	89.3	98	96
Mother	45	110	92.2	44	46
Father	47	120	102.3	115	114
Uncle	43	107	87.7	108	107
Wife	23	114	93.2	105	106
Reference Range		70 ~ 140	63 ~ 130	85 ~ 120	80 ~ 120

Based on the American College of Medical Genetics and Genomics (ACMG) guidelines for variant classification [14], the c.964 A > T variant has been classified as likely pathogenic (PVS1, PM2), supported by compelling evidence: in vitro expression studies have demonstrated that the mutation results in the loss of function; its absence in normal control populations, and prediction analysis from the bioinformatics tool MutationTaster (https://www.mutationtaster.org/) indicates a damaging effect.

### Analysis of other risk factors for venous thrombosis

All family members tested negative for the factor V Leiden mutation (G1691A), the prothrombin G20210A mutation, and antiphospholipid antibodies.

### TGA

In comparison to the control group, TGA of the patients’ plasma indicated that the proband displayed hypercoagulability, evidenced by heightened ETP (1520.8 ± 39.6 nM*min) and peak (382.3 ± 15.5 nM), along with decreased lagtime (1.4 ± 0.2 min) and ttPeak (3.4 ± 0.2 min). The plasma thrombin generation capacity of mother and father were normal. The TGA results of the mother, who carried a heterozygous c.964 A > T mutation, were also normal, due to a significant reduction in her factor II levels, as previously reported [12]. An unpaired Student’s t-test was conducted to compare four parameters between the experimental group, comprising the proband, father, and mother, and the control group. The analysis was performed using GraphPad Prism version 6.0. Figure 1C/D illustrated the thrombin generation profiles and specific parameters for each of the four groups.

### In silico analysis

Homologous sequence alignment results showed that p.Lys322 was not highly conserved across the homologous species. However, 43 out of the 143 amino acids deleted by p.Lys322stop were highly conserved among homologous species. The nonsense mutation (c.964A > T/p.Lys322stop) led to the deletion of 143 amino acid residues, causing the disulfide bond sites Cys247-Cys430, the glycosylation site Asn192, and P1-P1’ (Arg393-Ser394) to be lost. Additionally, this mutation disrupted three β-sheet structures and eliminated three α-helical structures in comparison to the original protein conformation (Fig. 3A).

### In vitro expression study of transfected HEK293T cells

**Fig. 2:**
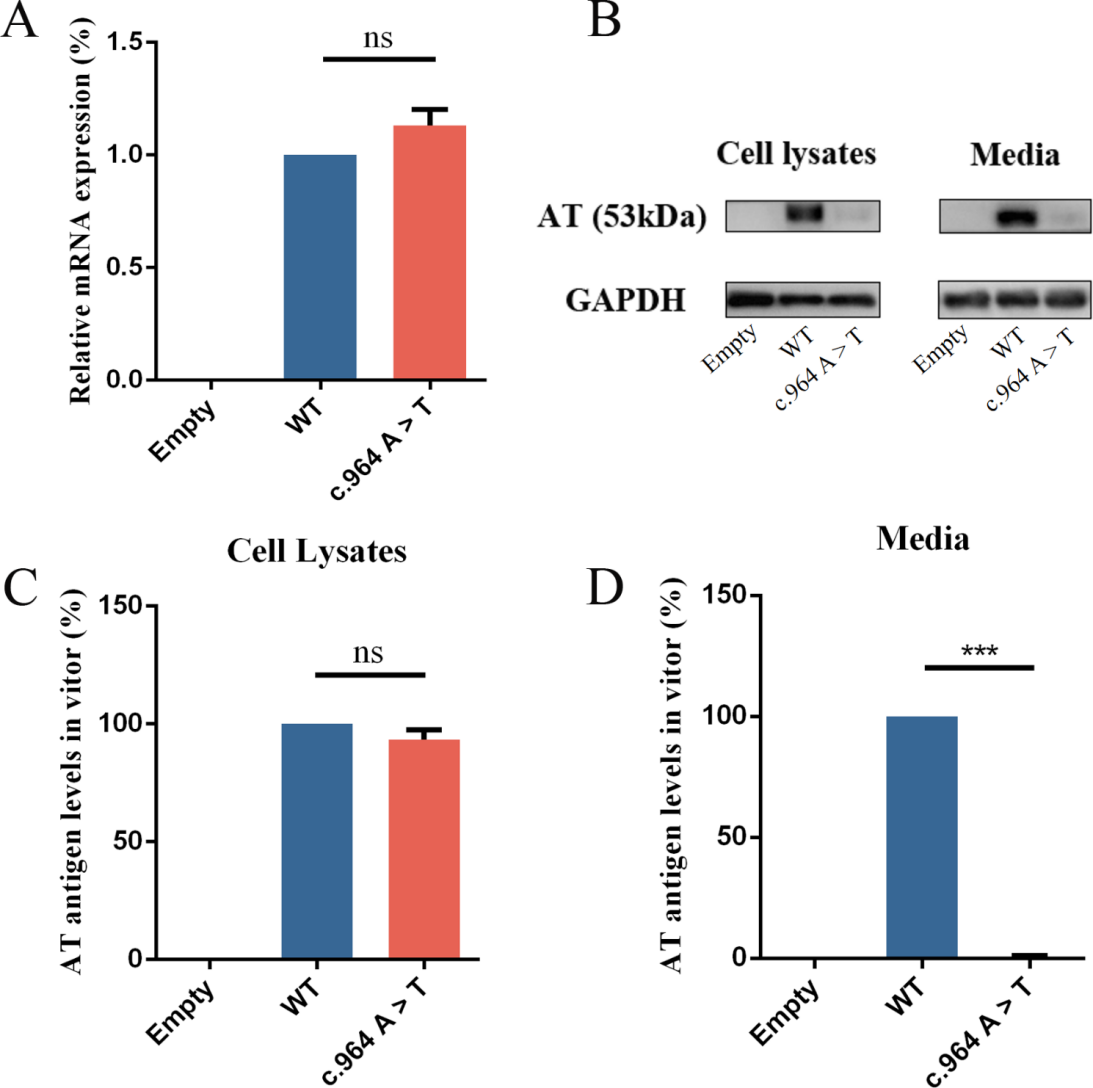
Variations of in vitro expression levels. (**A**) Relative quantification of mRNA levels in three recombinant expression vectors using the 2-ΔΔCt method, with normalization based on the expression levels of the GAPDH. (**B**) WB analysis of transfected HEK293T cell lysates and media. The levels of AT and GAPDH proteins were semi-quantified by SDS-PAGE. The quantification of AT protein in transfected HEK293T cells (**C**) and in culture media (**D**) was performed using ELISA to determine protein levels

**Fig. 3:**
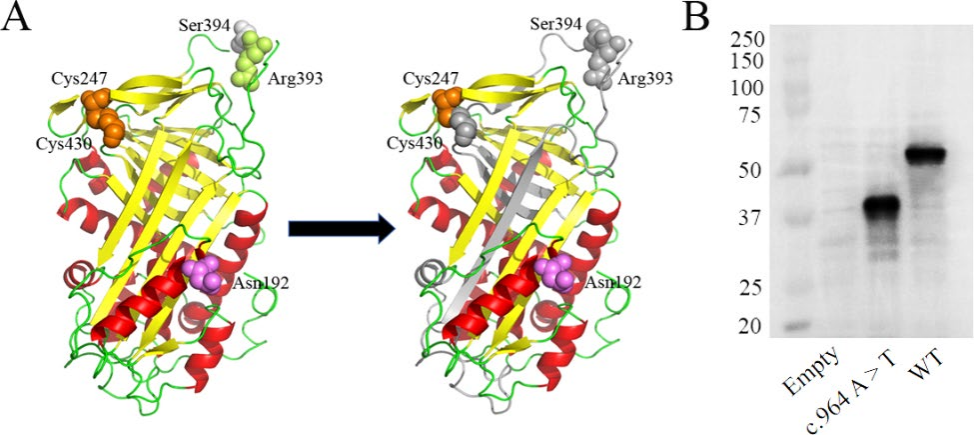
Model analysis diagrams. (**A**) Ribbon diagram of AT wild type and mutant type, the missing region of the AT structure in the mutant type is represented in gray. (**B**) WB analysis of transfected HEK293T cell lysates

**Fig. 4:**
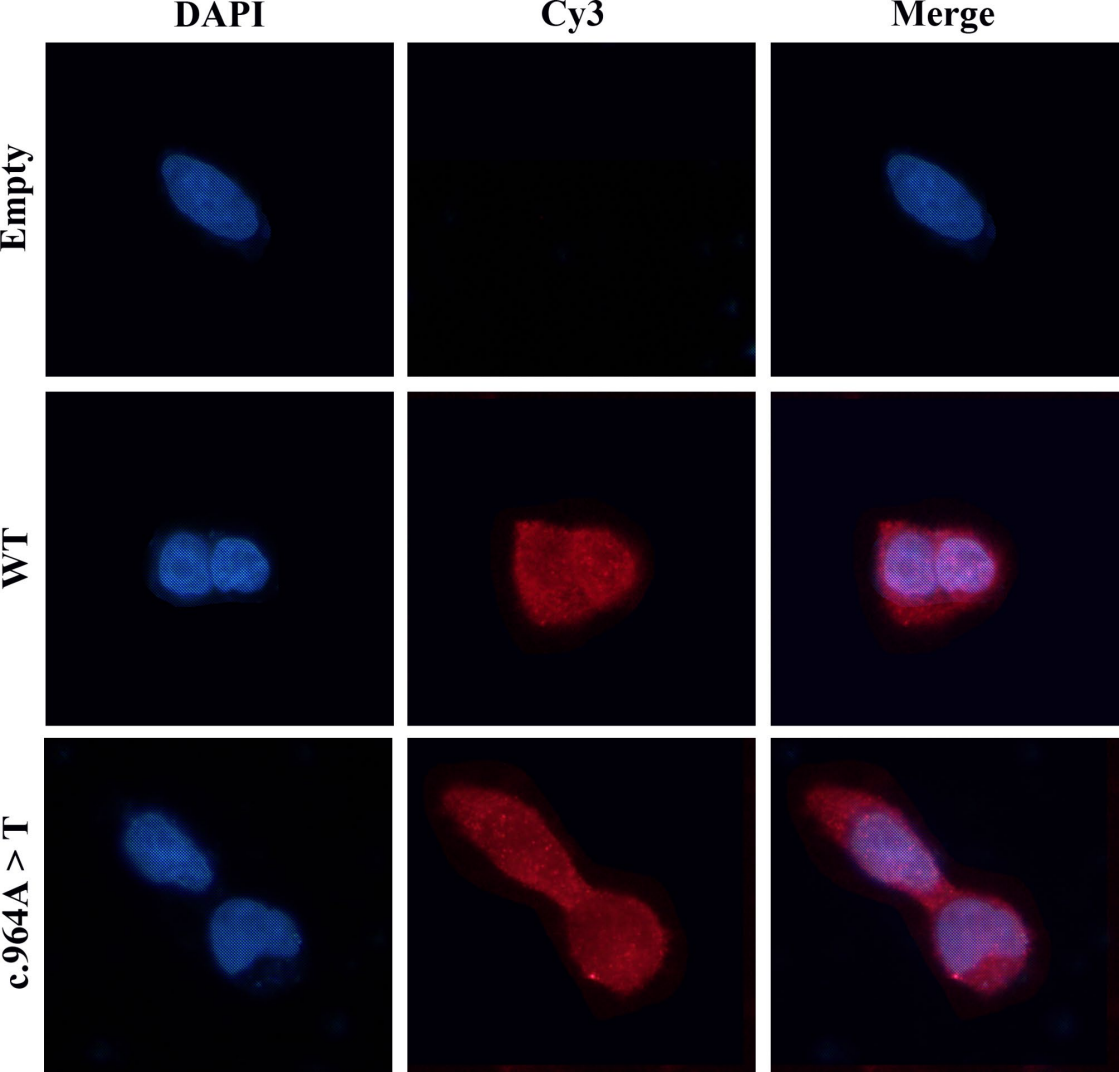
IF staining of empty vector (Empty), wild type (WT), and c.964 A > T. The nucleus is visualized with blue fluorescence; the AT protein is visualized with red fluorescence

The empty vector, wild-type, c.964 A > T (p.Lys322stop) plasmids were designated, labeled as Empty, WT, and c.964 A > T, respectively. All plasmids were transfected successfully with similar efficiency. The mRNA levels were assessed via RT-qPCR in HEK293T cells following plasmid transfection. As illustrated in Fig. 2A, cells transfected with c.964 A > T plasmid exhibited normal AT mRNA expression. The GAPDH expression remained constant across all groups. The ELISA examination of the cell lysates (Fig. 2C) indicated that the antigen levels was 93.33% ± 2.40% for c.964 A > T lysates. Interestingly, the analysis of the culture media unveiled that the level of c.964 A > T antigen was so low as to be undetectable (Fig. 2D). Protein immunoblotting analysis of plasmid-transfected cell concentrate medium showed clear bands of AT-WT at 53 kDa, whereas the bands at c.964 A > T were almost absent. In cell lysates, the AT-WT allele exhibited prominent bands at 53 kDa, while the c.964 A > T mutation displayed a distinct band at 35 kDa (Fig. 3B). As anticipated, no bands were observed in the media and lysates containing the empty plasmid. The results are presented as mean ± standard deviation (*n* ≥ 3). Transfected HEK293T cells were stained with Cy3. Subsequently, IF microscopy was used to investigate the existence of residual and/or abnormal intracellular AT proteins (Fig. 4). The AT-WT and AT-c.964 A > T proteins were both well distributed in the cytoplasm.

## Discussion

In the present study, we reported a patient with VTE. Laboratory analyses unveiled that the proband had only 50% AT: A (normal range: 85%~120%) and 49 mg/L AT: Ag (normal range: 80 ~ 120 mg/L). Genetic screening identified a point mutation in the *SERPINC1* gene of the proband, c.964 A > T/p.Lys322stop, inherited from his mother. Subsequently, the proband was diagnosed with hereditary type I AT deficiency.

The majority of mutations within the *SERPINC1* gene manifest in a heterozygous state. Patients with heterozygous type I AT deficiency are predisposed to developing VTE in youth, and childhood may also experience VTE triggered by specific acquired factors (such as surgery or trauma) [15]. Patients carrying nonsense mutation prone to occur recurrent DVT. The mutation of p.Asn107stop has been correlated with recurrent DVT and cerebral venous sinus thrombosis [8]. The mutation (p.Leu134stop) can lead to idiopathic popliteal DVT of the left leg [16]. The mutation (p.Arg197stop) can lead to recurrent DVT, lower limb venous insufficiency, varicose vein excision, leg ulcers, and a family history of VTE [17]. Similarly, the mutation p.Glu271stop has been found to be associated with recurrent DVT, cerebral artery thrombosis and pulmonary embolism [18]. Additionally, the mutation Gln337stop has been associated with an increased risk of pediatric cerebral sinus venous thrombosis [19]. In the current investigation, a mutation (c.964 A > T/p.Lys322stop) of *SERPINC1* was discovered in a Chinese youth suffering from venous thrombosis in the lower extremities and acute pulmonary embolism infarction. Therefore, we hypothesized that the c.964 A > T mutation was primarily responsible for the type I AT deficiency and consequent VTE in the proband. The TGA conducted on PPP from the proband further validation for our speculation. The proband exhibited an elevated potential for thrombin formation compared to healthy controls.

The most common point mutations in the 7 exons of the *SERPINC1* gene occur in exon 2 and exon 5, accounting for 34% and 26% of all point mutations, respectively [8]. Notably, the mutation c.964A > T just occurred on exon 5. Upon analysis of multiple sequence alignments, it was revealed that 43 of the 143 amino acids deleted by p.Lys322stop were highly conserved across homologous species, suggesting their crucial role in maintaining the structure and function of AT protein during biological evolution. The substitution of Lys322 with a premature stop codon (UAG) leads to the deletion of 143 amino acid residues, disrupting three β-fold and resulting in the absence of three α-helical structures. Its also causes the loss of a disulfide bond site, a glycosylation site, and a P1-P1’ site. These alterations are likely to compromise the stability of the mutant protein, making it susceptible to degradation and leading to a reduction in plasma AT levels. Hence, all above evidences pointed toward p.Lys322stop as a pathogenic variant.

Nevertheless, the most authoritative criterion for evaluating the clinical pathogenicity of mutations remains in vitro expression investigations of recombinant proteins in human cell systems. Therefore, to further investigate the pathogenic mechanisms of AT defects caused by the mutations, we constructed empty (Empty), wild-type (WT), and c.964 A > T (Mt) plasmid vectors, which were subsequently transfected into HEK293T cells for expression analysis. The analysis of cellular transcription levels indicated that the mutation will not affect the synthesis and/or stability of mRNA. The WB analysis showed that AT-Mt was detected clearly at 35 kDa, while its was almost undetectable at 53 kDa in cell lysates. In addition, AT-Mt was almost undetectable at both 35 kDa and 53 kDa in the media, suggesting that AT-Mt may compromise the secretion capacity of mutant AT proteins. Furthermore, the results of quantification of intra- and extracellular AT protein were consistent with WB. The results of the AT: A levels utilizing the ELISA assay for the conditioned cell lysates of AT-Mt and media AT-Mt were 93.33% ± 2.4% and undetectable, respectively. The IF experiments also demonstrated the normal expression of AT-Mt in the cytoplasm.

Nonsense mutations leading to premature termination codon (PTCs) tended to occur with nonsense-mediated mRNA degradation or the production of truncated proteins. The PTCs induced by AT-Mt in this study occurred in exon 5. The mRNA level of AT-Mt, assessed by quantification, was nomal. The AT-Mt with a clear band of Mt being observed on 35 kDa-sized transblotting membranes in lysates, the corresponding AT-Mt protein was also detectable in lysates through ELISA/IF with high sensitivity, while the AT-Mt protein was undetectable in the culture media. These findings suggested that intracellular retention would eliminate the aberrant AT-Mt, leading to truncated Mt proteins not being detected in the medium.

It should be noted that there are some limitations in our current research. While we utilized cell models to explore the impact of the *SERPINC1* variant on AT levels, our study lacked in vivo animal experiments, which would have provided further insight into the mechanism of this genetic variant’s effect on AT levels.

In this study, we have elucidated the mechanism underlying the reduction in AT levels caused by the AT-K322*. AT-K322* results in the production of a truncated protein, which in turn impairs the secretion of AT, leading to AT deficiency. Our findings shed light on the molecular pathogenesis of thrombophilia associated with disease-causing variations in *SERPINC1*.

## Electronic supplementary material

Below is the link to the electronic supplementary material.

Supplementary Material 1

## Data Availability

The datasets generated for this study are available in online repositories. The names of the repository/repositories and accession number(s) can be found at: https://submit.ncbi.nlm.nih.gov/subs/?search=SUB14885133.
