# Supplementary material for: Clinical and functional characterization of p.Lys322stop variant in the SERPINC1 gene causing severe thrombophilia
Source: Orphanet J Rare Dis. 2024 Dec 20;19:480. doi: 10.1186/s13023-024-03498-y (PMC11662841; doi:10.1186/s13023-024-03498-y)

**Name:** Serpin C1/Antithrombin Ⅲ Monoclonal antibody

**Size:** 53 kDa

**Brand:** proteintech

**Name:** GAPDH Polyclonal antibody

**Size:** 36 kDa

**Brand:** proteintech

**The original images of Cell lysates in Fig. 2B**


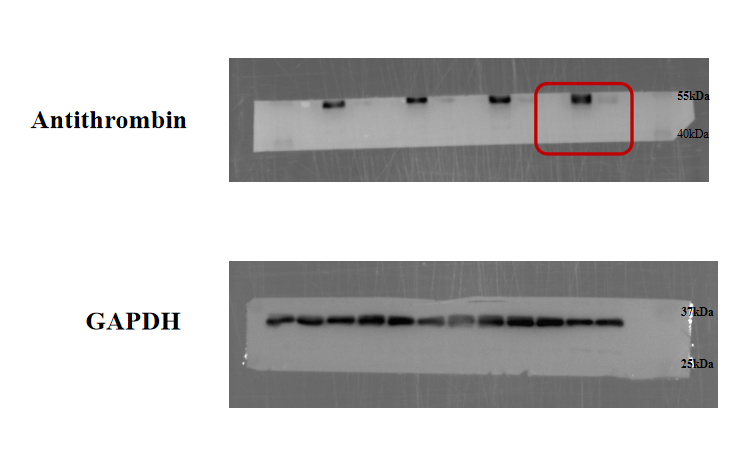


**The original images of Media in Fig. 2B**


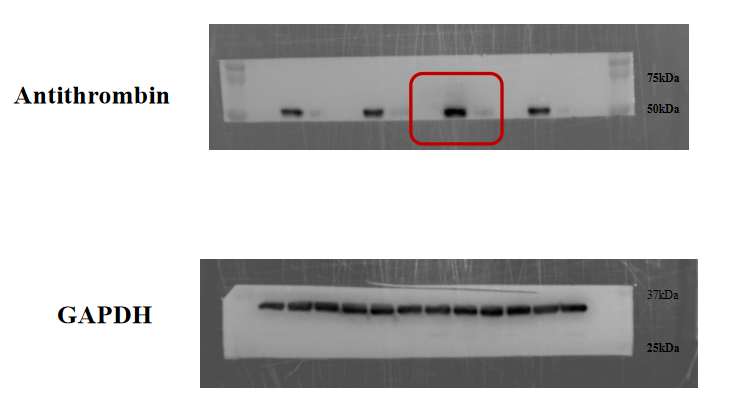

Supplement: Supplementary file 1 — Supplementary Material 1 [file 13023_2024_3498_MOESM1_ESM.docx]
